# A New Inquisition

**Published:** 1888-09-01

**Authors:** 


### The Hospital

A Weekly Institutional Journal of

### Science, Medicine, Nursing, and Philanthropy.

*For Hospitals, Asylums, Medical Practitioners, Students, Nurses, Families, Parishes, Congregations, and the Charitable Public.*

Vol. IV. No. 101.] [September 1, 1888.

### A New Inquisition.

In the days when faith was strong inquiry was keen. The Churchman who believed that his "credo" contained everlasting salvation in its substance, and that no other credo whatsoever enclosed such a germinal principle, was justified in a degree of zealousness which seems to us moderns to savour of fanaticism. Depth and strength of conviction are always associated with a keen and eager determination to protect and advance the cause believed in. It is because we believe in hospitals, and hospitals conducted on the voluntary principle, with all the earnestness of profound conviction that we watch with jealous eye any departure from the highest and worthiest principles of hospital management. No reasonable man will expect an impossible perfection in practice; every rightminded man will insist upon a perfect standard in principle, and will strive with all his might to approach that standard in details as nearly as may be possible.

Last week, under the title of "Hospital Finance and Economy," we commenced the publication of facts and statistics prepared for the Council of the Hospital Sunday Fund. In making their awards the Committee of the Fund required the representatives of one hundred and nine hospitals and fifty dispensaries to furnish them with detailed information respecting the cost per week of each occupied bed, the amount expended on each out-patient, and the average income and expenditure of each establishment during the last three years.

It is plain that, furnished with such data, the Committee were able to see quite clearly what hospitals were managed at a high rate of expenditure, what at a low, and what at a medium rate. They could not see more than this. The price of the article was given to them by each manufacturer, but not the quality. For a correct judgment of any man's work the quality must be taken in conjunction with the price. There are also, in dealing with a number of institutions, various local and other peculiar circumstances to be taken into consideration; and he who is just will always give to each of these its full modifying value. But when all these circumstances are duly allowed for, and others which may not be known, we are still compelled to the conclusion that the statistics of the one hundred and nine hospitals and fifty dispensaries cannot claim a verdict of entire approval from a candid critic. Here, for example, is a fact which must be absolutely astonishing to the average reader: The annual cost per occupied bed at —— Hospital is £180, whilst the annual cost at —— Hospital is £30. If a simple sum in arithmetic is worked out and one hundred and eighty is divided by thirty, it is seen that the cost per bed at the one hospital is exactly six times as much as at the other. In other words, it costs as much to keep one in-patient at the one institution as it does to keep six at the other.

Now this is too glaring! The most partial of judges cannot but admit that though there may be no positive dishonesty, there must either be criminal extravagance or such a degree of peculiarity of circumstances that an explanation should have been published along with the otherwise inexplicable facts.

It must not be supposed that this is an isolated contrast. True, it is the most striking; but there are others which approach it very nearly, and which but for it would be considered almost past belief. One such may be mentioned: The hospital at —— spends £176 per annum on each occupied bed, whilst the hospital at —— spends only £43. That is to say, it costs more to keep one in-patient at the former than it does to keep four at the latter. It will naturally occur to many to conclude that the hospitals which spend such extravagant sums on their patients must be wealthy endowed institutions, whilst those which practice such severe economy must be poor, struggling, voluntary charities. Nothing of the kind! Perhaps the most incredible fact of all connected with them is that all four are "supported by voluntary contributions."

The north and the south poles of hospital expenditure have thus been pointed out. Between these there is every degree of latitude. Anything like uniformity is conspicuous by its absence. Identity of expenditure is not to be expected, because it is not possible; but a reasonable degree of uniformity ought not only to be desired, but insisted upon. It may be right and just for the butcher to charge one shilling and fourpence a pound for the best steak at Kensington, although his confrère in Whitechapel only charges a shilling. But if the inhabitants of the western suburb were called upon to pay six shillings for the very same thing which could be purchased for one in the eastern, it is probable there would be a revolution in the kingdom of King Butcher even among the dames of Kensington. There are limits to the patience and good feeling of even the most amiable of hospital supporters, and it seems to us that those limits are distinctly within sight.

Practical readers naturally ask what is a reasonable standard of expenditure for ordinary hospitals? Guy's Hospital expends a little over £81 per bed per annum. We may therefore fairly conclude that £90 should ordinarily be abundantly sufficient for each occupied bed at other hospitals. Of the one hundred and nine hospitals which have furnished statistics, twenty-three spend less than this amount, whilst seventeen spend less than £80. But, on the other hand, there are no fewer than twenty metropolitan hospitals which spend £100 and upwards on each occupied bed per annum. All these, it must be borne in mind, receive help from the Hospital Sunday Fund. It would be both interesting and instructive to compare with this rate of expenditure the rates obtaining in the large provincial hospitals and in the hospitals of Scotland and Ireland. That we propose to do on some future occasion. For the present, attention is directed to the London medical charities.

We have to ask those hospital managers, whose institutions are such conspicuous targets for adverse criticism, what they propose to do? What steps do they conceive it desirable to take to retrieve their credit with the public? They cannot suppose that hospital supporters will approve or even condone their conduct. They ought not either to approve or condone it themselves. We do not charge any particular institution with dishonesty, but we do say that there is wrong-doing somewhere. There is a grossly extravagant waste of public money—a waste for which somebody must admit responsibility. Who is responsible? Is it the secretaries or the matrons, or the committees of the defaulting charities? We have no hesitation in answering these questions. It is the managers on whom the responsibility must ultimately fall. Their neglect or their incompetence has made the waste possible.

It is our desire to call the attention of hospital committees rather than of the uninformed public to the facts, in order that steps may be taken immediately to bring about the needed reforms. But it will be quite impossible either to blind the eyes or to silence the tongues of outsiders, if once the facts are made widely known. At the present stage we carefully refrain from criticising particular institutions by name, although their circumstances are known to us in detail. But if immediate steps are not taken to inquire into the causes of the extravagance with a view to their prompt removal, the public interest will demand that each offending institution shall be publicly proclaimed, and that the extent of its defaulting shall be made known. It is madness to suppose that hospitals will escape the critical inquisition of their enemies, even if their friends should agree to cover up their misdeeds. There are newspaper writers and self-constituted critics by the score who will only be too glad to ride into public notoriety on the back of hospital extravagance. They will not spare particular institutions or their managers, but, on the contrary, will be the more eager to begin their attacks because they can give point to them by dragging the names of individuals and hospitals into the light of day. Those managers whose institutions are in the defaulters' list can have no difficulty in recognising the fact. If they be in any doubt, we venture to suggest that the affairs of every hospital whose annual cost per bed exceeds £90, are ripe for inquiry. The question for the consideration of hospital authorities is whether they will take a hint from a source which has proved itself in a thousand ways to be loyal to the backbone to all well-conducted medical charities, or whether they will wait until a general movement of the public shall cover them with discredit and disgrace.

